# Toward the Identification of a Specific Psychopathology of Substance Use Disorders

**DOI:** 10.3389/fpsyt.2017.00068

**Published:** 2017-04-27

**Authors:** Angelo G. I. Maremmani, Pier Paolo Pani, Luca Rovai, Silvia Bacciardi, Icro Maremmani

**Affiliations:** ^1^V.P. Dole Dual Diagnosis Unit, Santa Chiara University Hospital, University of Pisa, Pisa, Italy; ^2^Association for the Application of Neuroscientific Knowledge to Social Aims (AU-CNS), Lucca, Italy; ^3^Department of Psychiatry, North-Western Tuscany Region, Local Health Unit (Versilia Zone), Viareggio, Italy; ^4^Social and Health Services, Cagliari Public Health Trust (ASL Cagliari), Cagliari, Italy; ^5^Department of Psychiatry, North-Western Tuscany Region, Local Health Unit, Massa, Italy; ^6^G. De Lisio Institute of Behavioural Sciences, Pisa, Italy

**Keywords:** psychopathology, addiction, SCL-90, heroin, alcohol, cocaine, substance use disorder

## Abstract

Addiction is a mental illness in which psychiatric conditions imply a prominent burden. Psychopathological symptoms in substance use disorder (SUD) patients are usually viewed as being assignable to the sphere of a personality trait or of comorbidity, leaving doubts about the presence of a specific psychopathology that could only be related to the toxicomanic process. Our research group at the University of Pisa has shed light on the possible definition of a specific psychopathological dimension in SUDs. In heroin use disorder patients, performing an exploratory principal component factor analysis (PCA) on all the 90 items included in the SCL-90 questionnaire led to a five-factor solution. The first factor accounted for a depressive “worthlessness and being trapped” dimension; the second factor picked out a “somatic symptoms” dimension; the third identified a “sensitivity–psychoticism” dimension; the fourth a “panic–anxiety” dimension; and the fifth a “violence–suicide” dimension. These same results were replicated by applying the PCA to another Italian sample of 1,195 heroin addicts entering a Therapeutic Community Treatment. Further analyses confirmed the clusters of symptoms, independently of demographic and clinical characteristics, active heroin use, lifetime psychiatric problems, kind of treatment received, and, especially, other substances used by the patient such as alcohol or cocaine. Moreover, these clusters were able to discriminate patients affected by addiction from those affected by psychiatric diseases such as major depressive disorder. Our studies seem to suggest the trait-dependent, rather than the state-dependent, nature of the introduced psychopathology dimensions of SUDs.

## Introduction

Substance use disorder (SUD) individuals show an incredibly high comorbidity concomitance with mental illness, especially with anxiety, impulse control, and mood-related disorders (1, 2). Moreover, the association with psychotic disorders is greater than what could be expected by chance (3–10).

The linkage between substance use (abuse or dependence) and mental illness is tricky. From a theoretical point of view, four possible explanations can be put forward: the first is that the manifestation of addiction may be facilitated by the presence of a mental disorder; the second is that SUDs elicit the onset of other mental disorders; the third is that the underlying causes of substance use and other psychiatric disorders may be the same; and the fourth is that factors linked to sampling, selection of instruments for diagnosis, investigation, and analysis could have led to an incorrect estimation of comorbidity. Even if the existing literature has explored the correlations between substance use and different areas of psychopathology, and put forward hypotheses about the mechanisms that trigger substance use and/or psychopathology, there is a broad gray, much less studied area dedicated to inquiring whether the symptoms often reported by SUD patients (especially symptoms related to mood, anxiety, and impulse control domains) have to be considered as merely a comorbidity, or whether they belong directly and intrinsically to addiction *per se* (11). This is critical because, besides the issue of chronology in dual diagnosis (which disease come first between addiction and another mental illness), the point is the need to understand the real nature of addiction by looking at the possible presence of a psychopathology that is exclusively related to the toxicomanic process.

Actually, some criticism became public about the classical model of psychiatric comorbidity in the field of SUDs that leads to a high frequency of association between the two disorders. This close association raises doubts about whether the two conditions are actually independent, especially when taking into consideration the overlap between biological substrates and the neurophysiology of the psychiatric symptoms and addictive processes that are related to addiction (11, 12).

Our V.P. Dole Dual Diagnosis research unit at the University of Pisa, Italy, has worked hard on this specific issue in recent years. It was difficult for us to believe that addiction *per se* was the only disease to have no specific psychopathology, and that all the symptoms and psychiatric clusters shown in SUD individuals had to be considered as merely manifesting “comorbidity.” The view we put forward is supported by analyses on diverse samples of SUD individuals, considering potential confounding factors that could be misleading in finding the right route for moving forward to the moment of identification of a specific psychopathology of addiction.

## Exploratory Factor Analysis on SCL-90 Questionnaires of Heroin Use Disorder (HUD) Individuals Entering Agonist Opioid Treatment: Identification of Dominant Factors

Taking into account the ineliminable factor of uncertainty in the correct classification of symptomatology—as being intrinsic to the addictive disorder or as due to comorbidity—it seems best to try to approach the psychopathology of addicts by starting from a low inference level—rooted in the symptoms expressed by patients—rather than starting from a pre-established syndromic level such as that of DSM nosography. In this foundation, it was a priority to identify, in SUD subjects, psychological/psychiatric dimensions drawn from the spontaneous association between various symptoms. We started by subtyping patients, using a sample of heroin-dependent patients, and working with their responses to the Self-Report Symptom Inventory (SCL-90) survey. The choice of using heroin addicts was in line with our conviction that opiate addiction is a paradigm for the study of SUD (13). We considered a sample consisting of 1,055 subjects, evaluated at their treatment entry and named agonist opioid treatment sample (AOT cohort). Data were stored in the University of Pisa dataset: an anonymous database collected for research and clinical purposes. For details regarding the AOT cohort, see Maremmani et al. (14).

Using an exploratory principal component factor analysis (PCA) of the SCL-90 questionnaire, a five-factor solution was found. Seventy-seven items with a >0.40 loading were retained. The items that had shown the greatest loading supplied the names of the single factors. The first factor corresponded to a depressive “worthlessness and being trapped” domain that accounted for 29.9% of the variance. The second factor identified a “somatization” dimension that comprised 4.2% of the variance. The third factor marked out a “sensitivity–psychoticism” dimension that included 3.0% of the variance. Panic symptoms led to the fourth factor, named “panic–anxiety,” accounting for 2.15% of the variance. Finally, the fifth factor reflected aspects of “violence–suicide,” which accounted for 2.0% of the total variance. Taken together, the five factors accounted for 37.8% of the variance shown by the items. Considering the highest *z*-scores obtained for each of the five SCL-90 factors (dominant SCL-90 factor), subjects were allocated to one of the five mutually exclusive groups. The group whose dominant was “worthlessness and being trapped” comprised 150 subjects (14.2%), the group with “somatization” as its dominant gathered 257 subjects (24.4%), the group showing “sensitivity–psychoticism” as its dominant included 205 subjects (19.4%), the group identified by “panic–anxiety” as its dominant numbered 235 subjects (22.3%), and the group whose dominant was “violence–suicide” group profiled a cluster of 208 subjects (19.7%). These five groups were sufficiently distinct and failed to reveal any significant overlap. All these patients showed positive scores in their dominant factors only, alongside negative scores in all the others, the only exception being a small number of patients whose dominant was “worthlessness and being trapped,” who showed a positive score for the “sensitivity–psychoticism” factor. A discriminant analysis confirmed these results, indicating a percentage of correctly classified “grouped” cases as high as 95.26%.

The “worthlessness, feeling trapped” dimension was the leading factor bringing together depressive, obsessive–compulsive, and psychotic symptoms. These feelings are frequently reported by SUD subjects at treatment entry, who talk of a feeling of being trapped in a corner, abandoned; they worry too much about difficulties, they feel guilty and report no sexual drive. Obsessive–compulsive symptoms focus mainly on patients’ doubts about their capabilities, the decisions they have to make, and their acts. Memory impairment and compulsivity are not present in any domain. Thought disorders consist of feeling alone even at moments spent together with other individuals. These subjects describe a feeling of inferiority, show interpersonal sensitivity (they are easily hurt), report phobic anxiety (they do not like staying alone), and “often feel nervous and upset.” This factor is basically made up of obsessive, depressive, and psychotic features and is dominated by feelings of being trapped in a corner and uselessness. This dimension can be considered on the basis of the close linkage between SUD and mental disorders, in terms of the epidemiology of the two conditions, their psychological and neurobiological background and the shared psychopathological risk factors (15–22).

The second domain (somatization) is distinguished by a number of anxious and somatic elements, which could be typical of opiate withdrawal feature. These patients complain of back pain, muscle aches, weakness and tiredness, heavy legs and arms, paresthesia, and loss of sensitivity somewhere in the body. Cold shivers and hot flushes are possible too, even stomach ache and nausea. Getting to sleep is difficult and, as a rule, when sleep comes, it is disrupted. The second dimension, resembling an opioid withdrawal condition, may be related to a request for treatment.

The third factor (sensitivity–psychoticism) features psychoticism and sensitivity. Subjects think that people are looking at them and are talking about them, maybe organizing something against them. They think they are not respected because of their personal perspective. They believe that others do not sympathize with them or actually disapprove of their conduct. They feel uneasy or uncomfortable when they find they are being looked at by others, have to be in crowded places, or have to do things with others (e.g., eating). These behaviors can be considered as psychotic when patients feel sure that others influence, control, or read their thoughts. This dimension can be considered in the light of the self-medication hypothesis, due to the antipsychotic action of opioids (23–30) or is often related to the co-abuse of cannabis and stimulants (31–39).

The fourth factor (panic–anxiety) can be summarized as a fear of going around alone, traveling by train, bus, or subway (agoraphobia), fear of feeling sick or sensations of dizziness, and episodes of critical anxiety. Generalized fear is a feature, with the need to avoid activities or places in order to prevent acute anxiety. This dimension too may be involved in the overlap between anxiety and withdrawal symptomatology, as the two conditions share physiopathological and neurobiological features (40–42).

The fifth factor (violence–suicide) includes aggressiveness against others as well as self-direct aggressiveness. Rage, anger, and smashing things up are the key components of this domain. These individuals have a habit of arguing with others and showing high energy levels, together with returning to ideas about death. There is an extreme impulsiveness, which marks out the behaviors of SUD individuals, and should be assessed in light of the shared neurobiological background (the prefrontal cortex and limbic system) and risk factors (antisociality and drug-related lack of control) (43–51).

## Stability of the Psychopathological Profile of Heroin Addiction

The main issue related to the identification of a specific psychopathology of HUD was the need to determine whether this five-factor (compound) solution obtained from a sample of HUD individuals entering agonist opioid treatment was the direct outcome of the specific condition of these patients (at that time requesting pharmacological treatment), or whether it subsisted independently of the request for treatment. To better understand if we were facing a trait rather than a state nature for the proposed five factorial dimensions, we looked at confounding variables such as treatment choice, active use of substance, lifetime psychiatric problems, substance chosen, and major psychiatric conditions. To do this, we used two different cohorts of patients:

- Therapeutic community sample (TC cohort) (52)
- Major depression sample (MD cohort) (53).

### The Criterion of Staying Independent of the Choice of Treatment (e.g., Agonist Opioid Treatment versus Therapeutic Community)

To verify whether the five psychopathological dimensions identified in AOT cohort patients were in any case observable, independently of the treatment chosen, we compared the AOT cohort with heroin-dependent patients belonging to the TC cohort. Our expectation was that that these dimensions observed in a previous heroin opioid use disorder sample would be validated again in other samples of heroin addicts and that the severity of a subject’s psychopathology would be correlated with the specific treatment choice (54). The factorial analysis applied to the SCL-90 scores of individuals with opioid use disorder at a residential TC entry led to the same five-factor solution we pointed out in heroin addicts entering an AOT. Differences were observed in the two cohorts not only at a sociodemographic level, but also at a clinical one. Turning now to sociodemographic and clinical characteristics, patients entering TC or AOT did not present differences in gender or education, but they did differ in marital status, welfare benefits, employment, age, prior treatments, and heroin addiction length. It is important to notice the differences in the settings of the two treatments: AOT is a highly standardized program, usually centering on buprenorphine or methadone maintenance, distinguished by its scientifically proven effectiveness, regulated by clear operational procedures and guidelines (55–57), whereas TC is a more heterogeneous and less standardized residential program, which is subject to adaptations brought in to satisfy the needs of special populations (such as adolescents, women, and people affected by psychiatric comorbidity) and financial difficulties (58–61). The TC individuals selected for the study were recruited from 121 TCs set up in 8 different Italian regions. These TCs tended to differ from one another in the services they offered (pharmacological, including opioid agonist treatment, psychiatric social psychological and also educational, rehabilitative, work training, and so on), in the population target (e.g., gender-oriented, with a dual diagnosis, mother-and-child, with multiple-dependence), and in the treatment length (roughly speaking, it ranged from 3 months to 2 years) (52).

### Becoming Independent of Active Substance Use (Detoxified versus Non-Detoxified Patients)

We verified whether any differences emerged between the five SCL-90 dimensions previously identified through the application of PCA in comparing heroin-addicted patients who had already been detoxified (DTX) with those who were not yet detoxified (NDTX) from heroin at the time of entering a Therapeutic Community Treatment. Detoxified patients were defined as those who reported having already been detoxified and not requiring agonist opioid treatment during the first month of TC treatment. According to our results (62), it is striking that the DTX patients proved to be comparable with the NDTX ones in their demographic and clinical characteristics, whereas their situation was less severe, to a statistically significant degree, in all five SCL-90 dimensions of the psychological/psychiatric fundamental features. Looking at the five-factor solution, the greatest difference was found in the case of the somatic dimension, which was, in fact, the only dimension that successfully discriminated between the two groups of patients.

### The Inquiry Became Independent of the Presence of Lifetime Psychiatric Problems

To explore the possible impact of comorbid psychiatric conditions on the five psychopathological dimensions set out above, we verified whether heroin-addicted individuals with (PC-HA) or those without (NPC-HA) known lifetime psychiatric problems showed any differences in these five domains. We considered PC-HA patients on the basis of the previous presence of a psychiatric diagnosis, suicide attempts, psychiatric hospitalization, and psychiatric welfare benefits at treatment entry or psychiatric treatment prescription while in a therapeutic community. Considering the TC cohort, NPC-HA and PC-HA patients failed to show any differences in most of the demographic characteristics. Conversely, older age, longer history of heroin dependence, being female, and general pattern of discriminated social status were associated with a higher proportion of heroin addicts marked out by their more severe psychopathology (PC-HA).

The higher scores shown by PC-HA in the five psychopathological dimensions are in line with the predictably greater severity of psychological/psychiatric status in individuals with heroin dependence and psychiatric comorbidity. Anyhow, our studies show that even if the presence of lifetime psychiatric problems appears to be correlated with the severity of psychopathology (as documented by the SCL-90 scores), it does not seem to be related to its quality: actually, the “panic–anxiety” and “somatic” dimensions are the only factors that discriminated patients belonging to the PC-HA from the NPC-HA group. According to multivariate analysis, none of the other three domains were able to predict the allocation of subjects to the NPC-HA or PC-HA group, so their persistence as components of the SCL-90-defined structure of opioid use disorder may be considered independent of the presence of lifetime psychiatric problems (63).

### The Inquiry Became Independent of the Choice of Substance Used (Heroin versus Cocaine versus Alcohol)

We decided to explore the specific burden arising from the substance of abuse in identifying the psychopathological structure. We considered subjects affected by cocaine, alcohol, and heroin dependence according to a diagnosis based on clinical judgment, availability of the SCL-90 questionnaire, and an age of 18 years old or more, leading to the selection of a sample of 2,314 individuals (63). Patients with heroin, alcohol, or cocaine dependence showed differences in most of the demographic characteristics considered: differences emerged in the frequency of male gender, age of subject, living conditions, and marital status. Alcohol dependents were older, and they tended to live alone more often than heroin or cocaine dependents; heroin dependents were more frequently single and less frequently male than cocaine-dependent ones. In subjects with primary opioid dependence, those with cocaine as secondary substance of abuse showed a lower educational level than those who had alcohol as secondary substance of abuse, and in their group, the level of unemployment had to be calculated at a higher level than those who had any other secondary substance of abuse.

“Panic–anxiety” was the prominent psychopathological dimension of SUD individuals using heroin, cocaine, or alcohol as primary substance. By contrast, “violence–suicide” for the group of alcohol dependence subjects (11.4%) and “worthlessness-being trapped” for subjects with cocaine or heroin dependence (15.1 and 16.0%, respectively) turned out to be the least frequent dimensions. “Somatic symptoms” dimension was the only one showing statistically significant differences between groups—in particular, highlighting a stronger representation of heroin-dependent subjects than of cocaine-dependent ones; the “sensitivity–psychoticism” dimension was more strongly represented in alcohol-dependent subjects than in heroin-dependent ones; “violence–suicide” was more frequent in heroin-dependent than in cocaine-dependent or in alcohol-dependent subjects.

A positive association was confirmed using a multinomial logistic regression between both the “somatic symptoms” dimension and heroin dependence versus cocaine dependence and, once again, between both the “sensitivity–psychoticism” domain and alcohol dependence versus heroin dependence.

The further logistic regression analyzing cocaine versus alcohol as primary substance of abuse did not detect any significant association between the five domains and the primary substance of abuse.

“Panic–anxiety” was the most strongly represented psychopathological dimension for all of the three groups of patients. Group by group, the least frequent dimensions were “violence–suicide” and “sensitivity–psychoticism” for the group of patients with alcohol as secondary abused drug, “violence–suicide” for patients with no cocaine or with alcohol as secondary drug, and “worthlessness-being trapped” for patients with cocaine as secondary drug of abuse. No statistically significant differences were observed between the three groups in any of the five SCL-based psychopathological domains.

### Differentiation from Specific Psychiatric Psychopathological Dimensions [Major Depression (MD)]

Considering major psychiatric diagnosis, it is important to demonstrate that these five dimensions were able to differentiate SUD patients from other psychiatric patients.

Thus, we compared HUD patients with MD patients on the basis of the five SCL-90 dimensions already identified in heroin SUD patients. If our five dimensions are directly correlated with heroin SUD, we would expect to find a higher prevalence of these dimensions in heroin-addicted SUD patients than in MD ones.

The sample consisted of the sum of the AOT cohort and MD cohort, reaching a total of 1,476 patients fulfilling the DSM-IV, DSM-5 criteria for heroin dependence, heroin SUD, or, alternatively, MD (53). Heroin SUD patients present a lower level of severity of psychopathological symptoms in general and a lesser severity of current symptoms with respect to MD subjects. However, the Worthlessness-Being trapped, Somatic Symptoms, and Sensitivity–Psychoticism dimensions are more strongly represented in heroin-addicted SUD patients than in MD ones. Although differences in age, gender, and severity of psychopathological symptoms were observed, according to SCL-90 criteria, the best predictor of being a heroin-addicted SUD subject remains a prominent psychopathology.

## Discussion

The results of our studies open the way forward to the possible identification of a specific psychopathology of heroin addiction and addiction *per se*. These five-factor dimensions appear to be different from the dimensions that are related to mental illness patients and remain stable no matter which confounding factors are considered, among those that are most pertinent when we are dealing with addicted patients.

When the choice of treatment is considered, the model of SCL-90 used by us showed the same five-factor solution for both TC and AOT groups of individuals. Looking at the association between the five psychologically and/or psychiatrically dominant groups and their allocation to TC or AOT treatment, the “violence–suicide” and “somatic symptoms” dominant dimensions were correlated with AOT. This result should be interpreted after taking into consideration the fact that AOT patients are better equipped to handle the features of violence and suicide, as well as showing a positive effect on somatic dimensions that may be closely related to withdrawal symptoms (30). Considering now other confounding factors, it is comprehensible that patients who still have a job are at their first request for treatment, are distinguished by a short addiction history, and are therefore likely to select a less stringent treatment program, such as AOT, which will lead to treatment having lower repercussions on their daily life.

Considering the active use of heroin, the “somatic dimension” is the only differentiating dimension between DTX and NDTX groups that may easily be explained on the basis of the features that this dimension comprises. The SCL-90 items included in this dimension correspond to a number of somatic complaints (e.g., back pain, muscle aches, cold shivers and hot flushes, disturbed sleep, and nausea) (14), which are usually part of the opioid withdrawal syndrome (64). In fact, the lower score shown by NDTX subjects for the somatic dimension can plausibly be attributed to the low or zero level of tolerance they show to opioids. Regarding the lower psychopathological severity shown by DTX patients in the other four SCL-90-based psychopathological dimensions, besides the effect of the anti-withdrawal treatment, which should never be overlooked (65, 66), it might result from the interruption of a disruptive addiction-related lifestyle, with its influences on the usual affective and cognitive parameters of patients, as well as from the changes in expectations arising from the implementation of a detoxification program.

Indeed, although a lower severity of the SCL-90 scores of DTX patients was observed in all the psychopathological dimensions than in the scores recorded for the NDTX ones, its degree was greatest in the “somatic” dimension, followed by the “worthlessness-being trapped” and “violence–suicide” dimensions, and was lowest in the “panic–anxiety” and “sensitivity–psychoticism” ones. Actually, the magnitude of the reduction in the severity of symptoms distinguishing the first three dominant SCL-90 factors may have allowed some of the DTX patients to show their highest scores in the dimensions least affected by detoxification, such as the “panic–anxiety” and “sensitivity–psychoticism” dimensions. This explanation is consistent with our findings on the easier resolution of the physiological symptoms of withdrawal compared with the psychological ones. One particularly instructive example is anxiety, which, together with other affective components of the withdrawal condition—those related to the reduction of dopamine tone and to the activation of the stress system—tends to persist longer after the interruption of heroin use (67–70). In this case too, however, the alternative possibility—that patients who have been less severely damaged on psychological and psychiatric grounds may have found it easier to interrupt heroin use before entering TC—should also be kept in mind throughout treatment.

When we considered the presence/absence of lifetime psychiatric problems, the differences that emerged were consistent with previous work in which the same topic was explored, but different investigative analyses were used (54) and they were applied to other populations of opioid addicts (10, 71–78), revealing that greater age, longer history of heroin dependence, being female, and a general pattern of discriminated social status were all associated with a higher proportion of heroin addicts, who were, moreover, showing a more severe psychopathology. Considering the specific five-factor structure, the “somatic dimension” was the discriminating feature in the group characterized by the presence or absence of lifetime psychiatric problems. One possible explanation takes into account the high percentage of somatic symptomatology that is observed both in the general psychiatric population (79–81) and in the dual diagnosis addict population (72, 82, 83). With reference to the “Panic–Anxiety” dimension, it is worth noting that the same cerebral circuits—e.g., the locus coeruleus (LC) noradrenergic system—are involved in panic attacks and opioid withdrawal (84–89). This psychopathological condition can be invoked to explain the association between active involvement in heroin and the “Panic–Anxiety” dimension. Symptoms such as anhedonia, irritability, amotivational status, and dysthymia are consistent with changes in the late-time mesolimbic dopaminergic, with the activation of the CREB/dynorphin pathway and the dumping of the dopaminergic tone (16, 90–92), and may help to explain the presence of “Worthlessness-Being trapped” features (93). The alteration in the dopamine system may lead to “Sensitivity–Psychoticism,” in which attribution of salience can be the consequence not only of a generalized improvement in dopamine tone (50, 94), but also of a more effective activation of k opioid receptors (92, 95). Finally, the “Violence–Suicide” domain could be linked with impulsivity, behavioral disinhibition, and reckless behaviors that are related to ventral orbital cortices and anterior cingulated dysfunction (96–100). The involvement of these brain regions has been observed in neuroimaging studies (101–106), such as those linked to the exploratory circumstances of decision-making or other neuropsychological assignments carried out by chronic SUD individuals (44, 99, 107, 108).

Our previous studies had the aim of investigating major HUD samples, considering HUD as the paradigm of addiction. Surely, substance of abuse works on different neuronal systems, nearly all of them leading back to the DA-ergic and opiate systems. In aiming to explain addiction *per se*, it was important to exclude another confounding factor—none other than the specific substance of abuse: heroin, cocaine, or alcohol, leading to the consideration that this structure is largely independent of the specific drug used. The differences that emerged were definitely those related to the specificity of action of the single substance abused: the “somatic symptoms” dimension, for instance, was better represented in heroin-dependent versus cocaine-dependent subjects, and the “sensitivity-psychotic” dimension showed a higher percentage in alcohol versus heroin groups. On the other hand, the greater association of alcohol-dependent subject with the “sensitivity-psychotic” dimension rather than the heroin-dependent group could be due to the presence of a pre-eminently psychotic aspect of alcohol withdrawal or to the progression of alcohol dependence *per se* (109–114). In considering the frequency of the five predominant psychopathological dimensions in patients with primary opioid dependence, the use of alcohol or cocaine as secondary substance of abuse does not lead to any significant difference; thus, the use of these additional substances appears to have no significant impact on these dimensions. As regards psychiatric severity, considering the full sample, the SCL-90 average scores seem to follow a decreasing order in four of the five psychopathological dimensions, with patients who have primary alcohol dependence showing the highest and those with cocaine dependence the lowest severity. In any case, the “panic–anxiety” and “somatic symptoms” dimensions were the only ones capable of successfully differentiating between groups of SUD individuals; the fact that the “somatic symptoms” dimension is able to differentiate alcohol from heroin dependence must be considered reasonable once we consider the burden of withdrawal syndromes in these two forms of dependence when compared with the symptoms of cocaine-addicted individuals, which are more closely related to alcohol or heroin withdrawal. Conversely, when considering the somatic symptoms related to the SCL-90 questionnaire, only mild somatic symptoms, or none at all, are associated with cocaine dependence (14, 64). It is trickier to understand the greater severity induced by cocaine dependence and alcohol dependence in relation to the “panic–anxiety” dimension. These two forms of SUD are associated with a high odds ratio for the presence of anxiety disorders, given that anxiety is considered an important element in the toxicomanic progression from use to dependence (1, 115, 116). It should be remembered that anxiety is a frequent consequence of cocaine use or intoxication, and also that in alcohol dependence anxiety emerges as a component of withdrawal. Moreover, we should take into account that alcohol withdrawal-related anxiety, unlike most of the other physical symptoms accompanying withdrawal, may last for months (117, 118). Persistent changes in the GABA and NMDA circuits associated with the development of tolerance and withdrawal could be at the basis of the long-lasting nature of anxiety-related symptoms (41, 119). It is therefore probable that the higher scores shown in alcohol-dependent patients, as the contribution made by the “panic–anxiety” dimension to the capacity to differentiate between dependence on different substances, highlight the weight of withdrawal-related anxiety in the daily lives of individuals set apart by different addictions. The “somatic symptoms” dimension, for instance, turns out to function as the best factor in differentiating between the three populations of drug-dependent patients.

Finally, we defined the capability of this five-factor solution to differentiate between HUD and MD patients. Some of the differences that have been found are, of course, related to the pathophysiological course of the illness. In fact, while addiction tends to have an earlier onset (11, 120), the depressive episodes that are clearly expressed occur more and more frequently as time goes by, especially the reactive ones that are due to stressful events encountered in life (121). HUD patients are more frequently males than MD patients. In particular, women are twice as likely as men to be depressed, while men tend to present a higher risk of substance and alcohol abuse disorders (122–126). In this way, we were also able to test the importance of differences in age and gender in limiting the importance of the psychopathological symptoms. Of fact of particular interest is that in differentiating heroin SUD from MD patients, the quality of the psychopathology encountered is more important than the severity of symptoms. Looking at this in a quantitative way, MD patients have a more severe psychopathology overall, but, from a qualitative perspective, four out of the five specific dimensions in the five-factor solution for psychopathology differentiate heroin addicts from depressed subjects. As regards the Panic–Anxiety dimension, it is important to consider the involvement of a specific cerebral circuit—the LC noradrenergic system—in the affective and physiological changes seen in heroin addiction, especially those due to withdrawal and protracted withdrawal, as well as those found in Anxiety disorders (84–89). The inability of this dimension to differentiate between heroin-addicted SUD and MD patients may be related to the transnosographic nature of anxiety-related symptoms, which may be considered rather common features both of addictive and depressive disorders. We found that in heroin SUD patients, depressive symptomatology remains the most important and frequent psychopathological aspect of heroin SUD. Moreover, this symptomatology is less closely related to suicidal ideation than in depressed patients. Of the five dimensions in question, “worthlessness-being trapped” brings together depressive, obsessive–compulsive, and psychotic symptoms. It mainly reflects a depressive dimension distinguished by depressed mood, feelings of uselessness, being trapped in a corner, sad, abandoned, with no interest or goal, and unreasonably consumed by difficulties as well as feelings of guilt and experiencing a low or zero sexual drive. These symptoms identify an overall condition of depression, but it is critical to understand which kind of depression we are looking at. Depression can be due to the use of substances (e.g., opiates, benzodiazepines, and alcohol) or can be primary (in that case authorizing a dual diagnosis when it occurs in heroin-dependent patients). Hypothetically, and this is our view, a specific depression peculiar to heroin addiction may come to be recognized as a component of its own psychopathological structure.

It is not surprising that somatic symptoms can differentiate heroin-addicted SUD patients. This is in line with heroin addiction withdrawal symptoms that are linked with anxiety. Actually, a drug dependence-associated nature has been seen in 50% of agoraphobia cases, 40% of obsessive–compulsive disorders, 25% of social phobias, and 20% of panic cases (15, 127). More frequently, heroin SUD patients showed prominent sensitivity–psychoticism symptoms. This is in line with the observation that psychotic symptoms are reported in 40% of stimulant abusers (128, 129), and in half of the more chronic cocaine users (130, 131). In addition, we have demonstrated that, taken together, the sensitivity and psychoticism dimensions were linked with younger heroin SUD patients, whereas older subjects who had HUD showed higher values for the somatization and worthlessness-being trapped symptomatology (14). Finally, it seems of particular interest that MD patients were distinguished by suicide ideation, which, in heroin SUD patients, constitutes a dimension unrelated to the depressive one. In general, guilt and suicidality are a psychopathological substrate that is typical of a depressive state. In heroin SUD patients, their guilt appears to be more strongly linked with feelings of being trapped and with a consequent sense of worthlessness.

## Limitations

SCL-90 questionnaire was used to highlight the psychiatric and psychological profiles of individuals belonging to both samples. Symptoms were collected on subjective perceptions (self-administration). It is important to notice that each sample was collected from several different outpatient clinics or care units—a circumstance that led to some difficulties in obtaining an “objective” evaluation. In any case, having a self-driven questioner makes it possible to investigate symptoms from a dimensional perspective instead of using a group of several interviewers—a feature that inevitably leads lead to a non-uniform interviewer-related objective rating. It must, surely, be taken into account that some individuals may hide some symptoms, either voluntarily or involuntarily. In the end, due to the lack of any observer-related “objective” evaluation, caution is needed in interpreting these results.

Using questionnaires to check the tendency of patients to lie is bound to function as a factor that helps to ensure the fairness of our data.

The individuals involved in our study were without any formal psychiatric diagnosis. It is important to know that in Italy a diagnosis is formulated at a late stage of treatment, either in addiction facilities or local units. The impact of psychiatric problems cannot be discriminated by using the SCL-90 questionnaire, and, surely, we cannot say whether or how strongly the profiles identified may correlate with a specific diagnostic criterion. Of course, a formal and objective psychiatric diagnosis would have to distinguish between subjects who have and those who do not have a significant psychiatric condition.

Another limitation in using the SCL-90 scale is that it was administered at treatment entry only; the selection of one single time means highlighting only that specific moment in the life of a heroin addict; we certainly know that some symptoms may vary in accordance with the different stages of the disease, whereas some may improve or at least be reduced in their intensity due to a specific kind of treatment leading to a sort of underweighting or overweighting within our sample.

Moreover, the TC cohort and the OAT sample show differences in several factors (especially age and length of drug dependence). First, diagnosis in the two samples was inevitably differentiated; considering the DSM-based criteria applied in the OAT sample and the clinically based criteria used in the TC sample, bias should be allowed for. In this light, it is important to consider that clinicians have to use a careful approach to dependence diagnosis when they choose a TC program for their patients (considering the limitations on patients’ freedom in TC that lead to a high level of dropouts), which may entail the selection of patients who have a severe condition. On the other hand, one cannot exclude either the opposite situation in which individuals in the TC sample did not fully reflect the DSM diagnostic criteria. One consequence is that the magnitude of the measures of association used in our study could be underestimated or overestimated.

## Conclusion

Our studies shed light on a specific aggregation of psychopathological symptoms in cases of HUD—a fact that strengthens the feasibility of the five-factor solution. It is now possible to say that these aggregations of symptoms are stable regardless of demographic and clinical characteristics, kind of treatment chosen, active involvement in substance use, lifetime psychiatric problems, and the substance chosen. These results pave the way to the delineation of a trait nature in place of a state nature in the perception of the structure of these five-factor psychopathological dimensions of heroin addicts. Moreover, the results shown by implementing a rigorous comparison of different substances of abuse allow us to define addiction as a unitary condition.

## Author Contributions

AM and IM drafted the strategy of the review and the present manuscript. PP, SB, and AM reviewed the literature. SB and LR critically revised the article. All the authors read and approved the final manuscript.

## Conflict of Interest Statement

IM served as Board Member for Mundipharma, D&A Pharma, Reckitt Benckiser Pharmaceuticals, and Lundbeck. The other authors declare no conflict of interest. The sponsors had no role in the study design, in the collection, analysis or interpretation of data, in writing the manuscript, or in the decision to submit the manuscript for publication.
